# Assessment of the Successful External Cephalic Version Prognostic Parameters Effect on Final Mode of Delivery

**DOI:** 10.7759/cureus.16637

**Published:** 2021-07-26

**Authors:** Asalah S Felemban, Kholoud Arab, Asmaa Algarawi, Shahad k Abdulghaffar, Khadijah M Aljahdali, Malak A Alotaifi, Sumayya A Bafail, Tasneem M Bakhudayd

**Affiliations:** 1 Medicine, King Abdulaziz University Hospital, Jeddah, SAU; 2 Obstetrics and Gynecology, King Abdulaziz University Hospital, Jeddah, SAU; 3 Faculty of Medicine, King Abdulaziz University Hospital, Jeddah, SAU; 4 Pediatrics, King Abdulaziz University Hospital, Jeddah, SAU; 5 Faculty of Medicine, King Abdulaziz University, Jeddah, SAU

**Keywords:** amniotic fluid index, external cephalic version, body mass index: bmi, ksa:kingdom of saudi arabia, estimated fetal weight, parity

## Abstract

Aim

This study aims to evaluate the prognostic parameters of successful approach for an external cephalic version (ECV) procedure by considering the vaginal delivery as the optimal mode of delivery.

Methodology

A retrospective cohort study was done during June 2019 in the obstetrics and gynecology department at King Abdulaziz University Hospital. Data were collected between May 2009 and May 2019 and included all pregnant women who were candidates for the ECV. The primary objective was to assess the final mode of delivery in relation to the outcome of ECV followed by the secondary objective which was the prognostic parameters of the ECV procedure (body mass index, amniotic fluid index, parity, estimated fetal weight). Additional variables were maternal age, placental position and ethnicity.

Results

We have studied 86 pregnant women with ECV attempts the overall ECV success rate was for 46 women (59.7%). For the final mode of delivery, after a successful ECV procedure, 40 women (87%) whom had spontaneous vaginal delivery, in association to successful ECV, the prognostic parameters recorded the highest success rate were multiparous 35 (76.1%), body mass index between 25 and 29.9 (53.1%), women older than 30 years old (60.9%), gestational age between 37 to 39.6 weeks (56.5%). Posterior placental location 55.6%, estimated fetal weight more than 2500 (73.9%).

Conclusion

Successful ECV cases have recorded a significant increase in the incidence of spontaneous vaginal delivery and the outcome of ECV which is affected by many prognostic parameters such as parity, maternal age, gestational age, body mass index, amniotic fluid index (AFI) and estimated fetal weight (EFW).

## Introduction

Breech presentation occurs in 3% to 4% of all pregnant women at term [1-4]. The fatal outcome of breech deliveries is responsible for 5.8% of perinatal and neonatal mortality up to 28 days post-delivery among the term singleton deliveries in the Netherlands [5]. External cephalic version (ECV) has been proven to be effective in the reduction of the frequency of breech presentation after 36 or 37 weeks of gestation and consequently the number of cesarean deliveries for this condition [6,7] and is defined as the physical uterine maneuvering to change a breech positioned fetus to a vertex position by an expert obstetrician with ultrasound guidance [8]. The procedure is performed by applying pressure externally to the fetus through the gravid abdomen [7].

The success of ECV is extremely variable, ranging from 35% to 86% depending on different variables which affect the outcome of ECV procedure such as BMI, maternal age, amniotic fluid index, gestational age, parity, location of placenta, time in breech, presentation before the procedure and the ultrasonography [2,4,6,8-11]. According to a cohort study that was done they discovered these results: 2614 females (with average BMI 23.5 kg/m2) with breech and no previous cesarean section had undergone ECV, and 49% were effective. ECV has been more effective in multiparas (64%) than in nulliparas (40%) [5]. According to another study that was attempted at Portland, USA they discovered that the incidence of effective ECV in each maternal parameter was considerably different and it showed that the greatest parameters of effective ECV were BMI and maternal height, as maternal height rises, the chances of an effective ECV rises, while the chance of an effective ECV declines as BMI rises [4]. Providing that, literature had documented that ECV is significantly contributing in decreasing the rate of cesarean sections (45%, 65%, 55%-27%), respectively [2,3,12]. Even though, 25% of women in the US and 50% in Canada who were carrying breech fetuses had undergone cesarean delivery while they were able to deliver vaginally [2,3,12]. While as, in Jeroen Bosch Hospital the vaginal delivery rate has increased from 43% in 2013 to 71% in 2017 [11]. Few studies had efficiently assessed these parameters effectiveness in association to the final mode of delivery.

This study will evaluate the prognostic factors of successful approach for an ECV procedure by considering the vaginal delivery as the definitive mode of delivery and the optimal outcome.

## Materials and methods

We conducted a retrospective cohort study that was approved by the institutional review board (IRB), in June 2019 at King Abdulaziz University Hospital (KAUH) a tertiary center in Jeddah, the western region of Saudi Arabia, by using medical records (Phoenix) and archives.

From May 2009 until May 2019 under the department of obstetrics and gynecology, we included all pregnant women who were candidates for ECV, the eligibility criteria were pregnant women with a singleton fetus in breech position who were not having contraindications for ECV procedure. We excluded from our study women with multiple pregnancies or uterine anomalies, pregnancies complicated by placenta previa, premature ruptured membrane, antepartum hemorrhage and pre-eclampsia. The primary outcome was to assess the prognostic factors of successful approach for an ECV procedure by considering the vaginal delivery as the definitive mode of delivery and the optimal outcome. The secondary outcomes were prognostic parameters and its association with successful ECV in King Abdulaziz University Hospital.

Our primary predictors of interest were maternal body mass index (BMI), amniotic fluid index (AFI), parity, estimated fetal weight (EFW) and the outcome of ECV. Maternal BMI was calculated using the equation (BMI = kg/m^2^) which was measured at 36 to 41 weeks of gestation; pregnant women were assigned to a BMI category according to standard WHO classification. Amniotic fluid index (AFI) was calculated by adding the anteroposterior diameters of the largest empty fluid pocket (no fetal parts or umbilical cord) in each quadrant. The AFI was considered normal between 5 and 25 cm while readings less than 5 cm were considered as oligohydramnios and more than 25 cm as polyhydramnios. Additional variables under investigation were maternal age, height, placental position and ethnicity.

All statistical analyses were performed using Excel 2019 and SPSS version 21 (IBM Corp., Armonk, NY). Univariate analysis was done by measuring the frequency and describing the data using measurements of central tendency and standard deviation. Bivariate chi-squared analysis was carried out on the prognostic parameters findings with the outcome of ECV procedure and its association with final mode of delivery whether it was vaginal or cesarean section. T-test was also used to calculate the statistical significance between the outcome of ECV and maternal age, estimated fetal weight and the pregnant women body mass index. P-value <0.05 was considered to indicate statistical significance. The prevalence was given in percentage with 95% confidence level.

## Results

We have studied 86 pregnant women with ECV attempts that were made between May 2009 and May 2019. Of whom, 10 were rolled out as their files were inactive.

Accordingly, 77 ECV attempts were analyzed. The primary outcome was to evaluate the final mode of delivery among pregnant women with successful ECV attempts. In which, the overall ECV success rate was 46 women (59.7%) and the failure rate was 31 women (40.3%).

For the final mode of delivery 50 women (64.9%) had spontaneous vaginal delivery, 18 women (23.4%) had elective cesarean delivery and nine women (11.7%) had an emergency cesarean delivery as shown in Table 1.

**Table 1:** ECV outcome and the mode of delivery. ECV: external cephalic version; SVD: spontaneous vaginal delivery; ELCS: elective cesarian section; EMCS: emergency cesarian section.

Mode of delivery	SVD 64.9% (n=50)	ELCS 23.4% (n=18)	EMCS 11.7% (n=9)
Success = 59.7% (n=46)	87% (n=40)	8.7% (n=4)	4.3% (n=2)
Failure = 40.3% (n=10)	32% (n=10)	45.2% (n=14)	22.6% (n=7)

	SVD	ELCS	EMCS	Total
Successful ECV	40	4	2	46
Failure ECV	10	14	7	31
Total	50	18	9	77

Secondary outcome was to assess the effectiveness of the prognostic parameters with successful ECV procedures. For the gravidity status, there were 25 nulliparous (32.5%) and 52 multiparous (67.5%). Of these, 71 cases (92.2%) admitted from antenatal ward and 6 (7.8%) cases from the emergency department. The mean maternal age was 30.195 (SD = 5.3362) with a maximum age of 42 and a minimum of 20, there was a statistical difference between the maternal age and the outcome of the ECV (P-value = 0.028). The mean BMI of total ECV was 30.48 (SD 5.188) with a range that equals 23-44. The mean gestational age was 37.160 (SD 2.82), estimated fetal weight mean was 2686 g (SD 748.53). Ultrasonography exhibited multiple placental locations were 45 (58.4%) posteriorly followed by 21 (27.3%) anteriorly other locations accounted for 15% (anterior-fundal, posterior-fundal) and the data was not available for 2 (2.6%). All women who underwent ECV had an appropriate amniotic fluid index.

Our data had documented 12 different nationalities. The percentage of Saudi women was 62.3%, followed by Yemeni women with 13%, Palestinian with 5.2%, Sudanese with 3.9%, Pakistanis, Indians, Filipinos and Bangladeshi with 2.6%, Chadian, Burmese, Jordanian and Afghani with 1.3%. On assessing the pregnant women condition there were chronic history of diabetes mellitus in three women (3.9%), maternal hypertension in one woman (1.3%), gestational diabetes in three (3.9%) women and none of them had gestational hypertension. No uterine anomalies were recorded, four women (5.2%) who underwent ECV had previous abdominal surgeries and four others (5.2%) had a previous cesarean delivery along with normal fetal condition. Although no immediate complications from the ECV were recorded, 22 (28.6%) experienced failure of ECV, of whom, eight women (36.4%) required emergency cesarean delivery and 14 women (63.6%) had an elective cesarean delivery.

In the association between the prognostic parameters and the rate of successful ECV, the gravidity status was 11 for nulliparous with a percentage equals 23.9% and 35 (76.1%) for multiparous. Women with BMI between 25 to 29.9 had the highest successful rate of ECV (53.1%). Maternal age older than 30 years old rated 60.9% in comparison to women younger (39.1%). <37 weeks of gestation was 30.4%, >39 weeks was 13.0% and between 37 to 39.6 gestational weeks were the highest rate and accounted for 56.5%. In relation to the placenta location, pregnant women with Posterior placenta were documented with successful rate of 55.6%, followed by the anterior location 31.1%. For fetal conditions, the success rate was more with an estimated fetal weight of more than 2500 (73.9%).

## Discussion

This present study illustrated that the outcome of ECV and the final mode of delivery is statistically significant (P = 0.000) as ECV procedure documented a success rate in 59.6% of women, and the overall vaginal delivery rate after a successful ECV was 87% and the rate for both elective and emergency cesarean section was 13%. These findings are consistent with a study that was reported in Taif, Saudi Arabia that showed 84.1% of the women delivered vaginally and 14.5% of them underwent cesarean section after a successful ECV. It is suggested that ECV should be offered to all women with a breech presentation to minimize the danger of non-cephalic presentation at term and decrease the risk of cesarean section [13]. 

In our study, we found that ECV success significantly increases with maternal age older than 30. In contrast, ECV success rate decreases as maternal age become <30 (P = 0.028) although there could be other prognostic parameters that are affecting the success rate of ECV along with the maternal age such as BMI which was classified according to WHO classification and due to the expectation of a mean weight rise of 9 kg during pregnancy, we did our calculation by subtracting the expected mean 9-kg weight rise based on Burgos et al [14]. And as BMI was in the normal range and the gravidity status was multigravida along with >30 years of maternal age the success rate increases from 37.5% to 54.3%. The rate of success is proportionally related to both EFW and age. Pregnant women older than 30 years old and at 37-39.6 weeks of gestation their rate of successful ECV increases (58.3%).

Numerous studies have investigated the variables affecting ECV’s success rate; however, the majority of research varies significantly in terms of methodological quality and sample size. As well as maternal weight, BMI, amniotic fluid index, and estimated fetal weight were the factors most frequently used in previous studies. Both Kok et al. and Burgos et al. found that parity and AFI are predictive for successful ECV procedure. [14,15] Which in fact supports our result that reports a higher success rate (76.1%) for multiparous women in comparison to nulliparous (23.9%). However, all our data for AFI were within the normal range which is between 7 and 25 centimeters, these two previous studies suggested that AFI below 10 centimeters had an impact on the outcome of the ECV and therefore this may lower the success rate. [2,16,17] Furthermore, Shalev et al analyzed many variables and found a significant association between successful version and EFW [18] as our study showed EFW is related to the outcome of ECV and optimum EFW for a successful ECV was 2686 g.

BMI was also taken under consideration in our study and so many other studies [2,14,16,19], as they reported that women with normal BMI are more likely to have a successful ECV than women with greater BMI.

## Conclusions

In conclusion, successful ECV cases has recorded 59.6% of women. The results from this study demonstrated vaginal delivery rate after a successful ECV was 87%, While the rate of emergency and elective cesarean section was 13%.There is significant increase in the incidence of spontaneous vaginal delivery after ECV. Outcome of ECV is affected by many prognostic parameters such as: parity, maternal age (women older than 30 years old), 37-39.6 weeks of gestation, BMI within normal range, AFI within normal range and the optimal EFW 2686 g. It is suggested that ECV should be offered to all women with a breech presentation to minimize the danger of non-cephalic presentation at term and decrease the risk of caesarean section.
